# Rotigotine in the Long-Term Treatment of Severe RLS with Augmentation: A Series of 28 Cases

**DOI:** 10.1155/2011/468952

**Published:** 2011-01-23

**Authors:** Jana Godau, Daniela Berg

**Affiliations:** Hertie Institute for Clinical Brain Research, University of Tübingen Center of Neurology, Hoppe-Seyler-Straße 3, 72076 Tübingen, Germany

## Abstract

This structured clinical observation includes 28 patients with severe RLS, severe augmentation, and previously frustrating changes of dopaminergic treatment. All were switched from their current dopaminergic regimen to an individually adjusted rotigotine monotherapy; dosages were kept stable for 12 months. Follow-up exams were performed after 1, 3, 6, and 12 months. Severity of RLS symptoms (IRLS), augmentation (ASRS), depressive symptoms (BDI), and daytime sleepiness (ESS) were assessed at all visits. Median rotigotine dose was 4 mg. 27 of the 28 patients showed a major to complete reduction of RLS symptoms. IRLS and BDI scores (both *P* < .001), but not ESS scores, were significantly reduced. IRLS and BDI amelioration remained stable over the 12-month follow-up period. Augmentation occurred in only one patient. 71.4% suffered at least one mostly mild side effect; most common were increased appetite with compulsive eating (42.9%), application site reaction (28.6%), and nausea (14.3%). In the clinical setting, rotigotine seems to be valuable for the long-term treatment of patients with severe RLS and augmentation.

## 1. Introduction

Restless legs syndrome (RLS) with a prevalence of about 10% is one of the most common neurological disorders. It is characterized by an irresistible urge to move the legs and sometimes other body parts, which is accompanied by sensory leg discomfort, that occurs at rest and may be relieved by moving and follows a circadian rhythm with most pronounced symptoms at night [1, 2]. 

RLS symptoms may be effectively controlled using dopaminergic drugs. A number of studies suggest that regular (rr) and sustained release (sr) levodopa [3–5], pramipexole [6–9], ropinirole [10–13], and cabergoline [14–17] are useful for the short- and long-term treatment of RLS symptoms. All of these drugs share common side effects, including nausea, edema, and orthostatic hypotension, but are generally well tolerated. Another important side effect of dopaminergic medication is augmentation, which is defined as gain of symptoms regarding localization, time course and severeness while treatment may still be effective, leading to a gradual increase of daily dosages. Treatment is provided by either dose reduction or switch to another dopaminergic agent [1, 18]. Different studies have shown that augmentation occurs in up to 80% of patients treated with levodopa [1, 19] and in about 30% of patients treated with pramipexole [20, 21]. Usually, augmentation manifests within the first year of treatment, but also later in the treatment course augmentation may occur [22]. The pathophysiology of augmentation in RLS is only partly understood. Iron deficiency, loss of responsiveness of dopamine receptors in specific brain regions, continuous dopaminergic overstimulation, and also pulsatile stimulation contrasting the chronobiological regulation of endogenous dopamine have been discussed as potential sources of RLS augmentation [23]. 

Rotigotine as a transdermally applied D2/D3 receptor agonist is acting continuously and might therefore have the potential to reduce symptoms of augmentation [24]. Initially it had been developed for the treatment of early- and late-stage Parkinson's disease. Its effectiveness has also been proven for the application in mild and moderate RLS [25, 26]. In a one-week proof-of-concept trial, also the effectiveness for the treatment of severe RLS was shown [26]. A large randomized controlled trial including 341 patients suggested best effects at dosages of 1–3 mg/24 hrs [25]. 295 of these patients continued the medication in a 12-month open label extension trial. Although only 220 patients (74.6%) completed the study, data demonstrated good effectiveness, safety, and tolerability of rotigotine at a median dosage of 4 mg/24 hrs [27]. No patient in this cohort reported augmentation.

Due to the nature of clinical trials, very severely affected patients with augmentation were not eligible for these studies, as they could not be kept off medication for a 1–4-week washout phase prior to study inclusion. Here, we report data on a series of subjects affected that severely who were switched to rotigotine in the clinical setting.

## 2. Patients and Methods

A series of 28 patients with severe RLS, who were insufficiently treated under their current dopaminergic medication and additionally suffered severe augmentation, requiring termination of the current dopaminergic regimen, were included. All had a history of at least one prior change of medication due to augmentation. All of them were naïve to rotigotine, which was at that time in the approval procedure for RLS, meaning that application was generally off-label but allowed for individual treatment as ultimate alternative before initiation of long-term opioid treatment. 

Patients were switched to rotigotine monotherapy in the outpatient setting and were routinely followed after 1, 3, 6, and 12 months. The initial dopaminergic medication was fully terminated in one step and substituted at the same time by rotigotine 1 mg/24 hours. All nondopaminergic medication was kept stable. The rotigotine dose was adjusted individually for optimal effect within the first three weeks and then maintained on a stable dose for the whole follow-up period. First, a rapid up-titration to the minimum effective dosage was performed as follows: 1 mg/24 hours (Day 1, 28 cases), 2 mg/24 hours (Day 2, 20 cases), 4 mg/24 hours (Day 3, 12 cases), 6 mg/24 hours (Day 5, 3 cases), and 8 mg (Day 7, 1 case). Further, slow titration by 1 mg/24 hours (up or down) every fourth day was performed until the optimal effective dose (max. 8 mg/24 hours) was achieved. Additional regular release L-Dopa was allowed as medication on demand up to a dose of 200 mg/day during the titration phase, although the patients were asked to omit this medication if possible. Patients were instructed to take domperidone 20 mg tid during the up-titration if they experienced nausea (4 cases); in all cases nausea could be controlled and did not lead to termination of the drug. 

In a standardized interview, patients were asked for satisfaction with the treatment and for potential side effects including nausea, vomiting, edema, weight gain, increased daytime sleepiness, sleep attacks, orthostatic hypotension, dizziness, headache, application site reaction, augmentation, impulse control disorders, and “other symptoms” according to a standardized questionnaire used in our outpatient clinic. RLS symptom severity was assessed using the International RLS Study Group Rating Scale (IRLS) [28], augmentation severity was measured by Augmentation severity rating scale (ASRS) [29], depressive symptoms were assessed using Becks Depression inventory (BDI) [30], daytime sleepiness was assessed using Epworth sleepiness scale (ESS) [31]. Clinically significant improvement was defined as a decrease of >20% for each of the scales. IRLS scores of or below 15 of 40 points were considered as a marker for effective control of RLS symptoms [28, 32]. 

Statistical analysis was performed using SPSS 14.0 (SPSS, Chicago, IL). Descriptive statistics are given as mean and standard deviations or as percentages. Kolmogorov-Smirnoff-test was performed to test for normal distribution of baseline values. Dependent on the results being either parametric or nonparametric, testing was applied for comparative and correlation analysis. Results were assumed to be significant at *P* < .05. When applicable, Bonferoni *P*-value correction for multiple comparisons was performed for subgroup analysis.

## 3. Results

Twenty women and 8 men with a mean age of 65  ±  10 years (mean  ±  SD) were examined. 71.4% were diagnosed with idiopathic RLS and 28.6% with comorbid RLS associated with polyneuropathy (7 patients) and spinal lesion (1 patient). No patient suffered iron deficiency, although 10 patients received oral iron supplementation. The median serum ferritin level was 140 *μ*g/L (range 90–270 *μ*g/L; laboratory reference range 50–300 *μ*g/L). Median age at symptom onset was 46 years (range 14–73 years), and median disease duration was 16 years (range 2–64 years). All subjects suffered RLS symptoms throughout the day interfering with daily activities, and in all except one patient also the arms and/or the trunk were affected by RLS symptoms. In all subjects, these symptoms had developed after initiation of dopaminergic treatment. All patients had documented excellent response of RLS symptoms to dopaminergic drugs.

At the time of inclusion in the survey (baseline), all patients were treated with combined rr- and sr-levodopa in a median daily dosage of 500 mg (range 200–1200 mg). 20 patients were additionally treated with a dopamine agonist: 13 with pramipexole (median daily dose 0.7 mg, range 0.18–2.1 mg), 5 with ropinirole (median daily dose 2 mg, range 1–8 mg), and 2 with cabergoline (2 and 4 mg). 

All patients were switched from their current dopaminergic regimen to a rotigotine monotherapy as indicated above. The dosage was adjusted individually for the optimal effect within the first three weeks and was then kept stable for the whole observational period.

Of the total of 28 patients included in the survey, 4 patients (14.3%) discontinued the drug within the first month and were therefore excluded from further followups: one because of lack of effectiveness and three because of side effects (see below). The median rotigotine dose for optimal effectiveness was 4 mg/24 hrs (range: 2–8 mg/hrs).

### 3.1. Effect on RLS Symptoms

**Figure 1:**
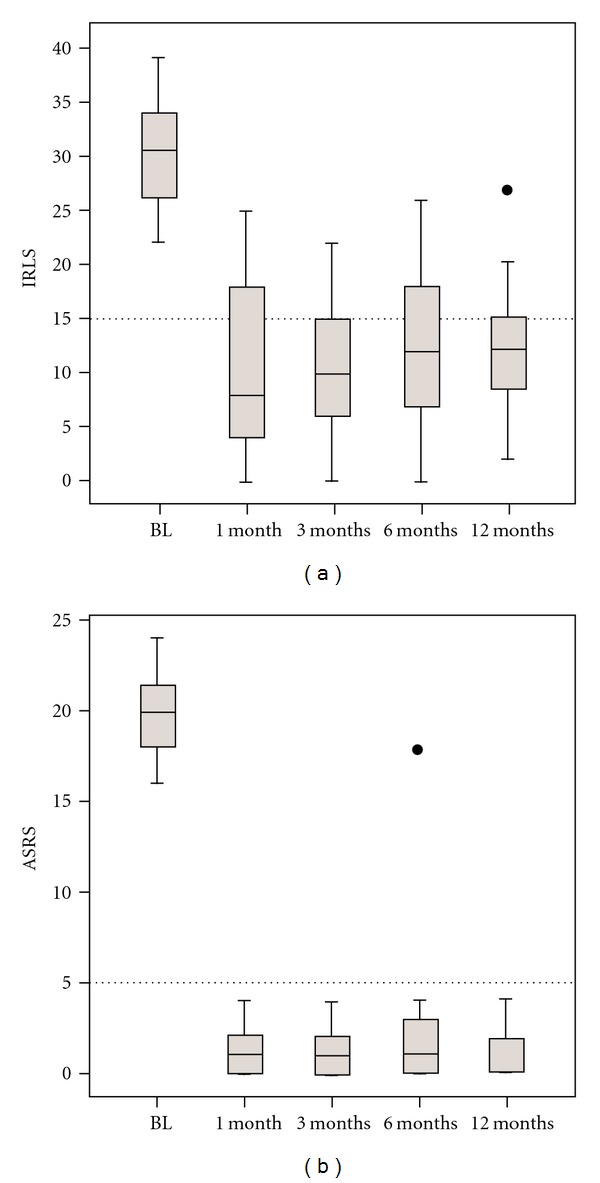
Serverity of RLS symptoms and augmentation. BL: baseline, IRLS: International RLS Study group rating scale, mo: months. (a) International RLS Severity rating scale IRLS. Values below the horizontal spaced line indicate fully effective treatment (IRLS  <  16 points). (b) Augmentation severity rating scale (ASRS).

Mean IRLS score was 30  ±  4 at baseline under the combined dopaminergic medication before switching to rotigotine. It decreased to 9  ±  7 after 1 month (*P* < .001). The followups at 3, 6, and 12 months showed no significant increase of mean IRLS scores compared to the assessment after the first month (Figure 1(a)). Twenty-seven of 28 patients showed a clinically significant (>20%) improvement of RLS severity after one month. IRLS reduction below a score of 15 points was achieved in 70.4% of the patients after one month, and in 56.6% after 12 months. Of the 24 subjects who completed the study, 1 was “not satisfied”, 13 patients were “very satisfied”, and 10 were “fully satisfied” with efficacy of rotigotine in the control of RLS symptoms after the 12 months treatment period.

### 3.2. Effects on Augmentation

Mean ASRS was 19.9  ±  2.4 points at baseline. After 1 month, signs of augmentation were not present in any of the patients, and mean ASRS was 1.2  ±  1.4 points (*P* < .001). Mean ASRS did not show a significant increase during the 12-month follow-up period (Figure 1(b)). Augmentation occurred in one patient and led to discontinuation of the drug after 6 months (ASRS 18 points).

### 3.3. Effects on Depressive Symptoms

**Figure 2:**
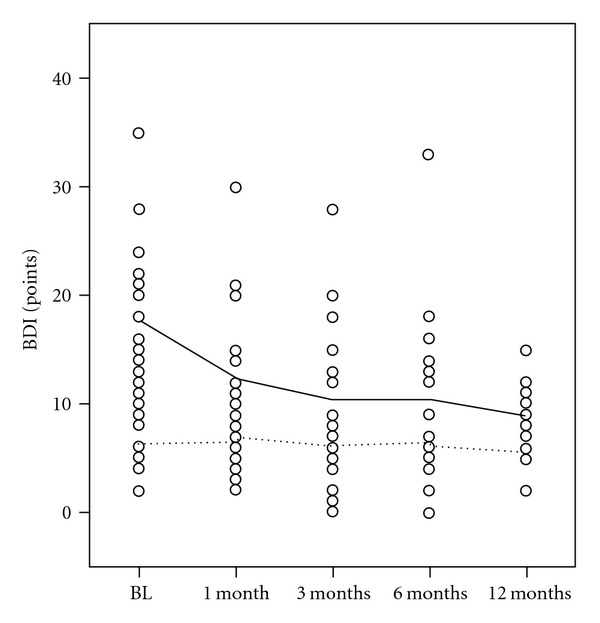
Severity of depressive symptoms. BDI: Becks Depression Inventory. BL: Baseline. Mo: months. Full line: Mean BDI for depressed RLS patients. Spaced line: Mean BDI for RLS patients without depression.

Mean BDI score at baseline was 13  ±  6 points. 67.9% of the patients suffered major depression according to DSM-IV criteria. In this subgroup, mean BDI score at baseline was 18  ±  7 points, compared to 6  ±  3 points in the nondepressed group. In the latter group BDI scores did not significantly change during follow-up examinations (Figure 2). In the group of depressed RLS patients, BDI scores were significantly reduced compared to baseline after three (10  ±  7, *P* = .016), six (10  ±  7, *P* = .014), and 12 months (9  ±  4, *P* = .002), with the most pronounced effect after 12 months. 52.6% of depressed patients showed a clinically significant BDI score reduction. Reduction of BDI scores did not correlate with IRLS reduction.

### 3.4. Effects on Sleepiness

**Figure 3:**
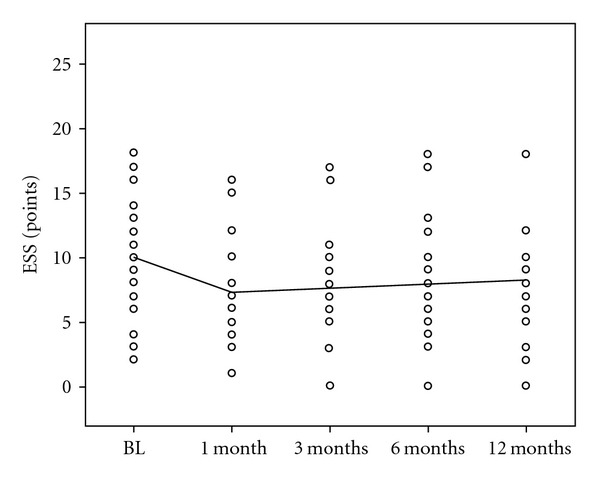
Daytime Sleepiness. BL: Baseline. ESS: Epworth Sleepiness Scale. Mo: months.

Mean ESS score at baseline was 9  ±  4 points. After 1-month followup mean ESS scores improved slightly, but not significantly (7  ±  4, *P* = .26). At the follow-up examinations after 3, 6, and 12 months ESS increased back to baseline values (Figure 3).

### 3.5. Side Effects

**Figure 4:**
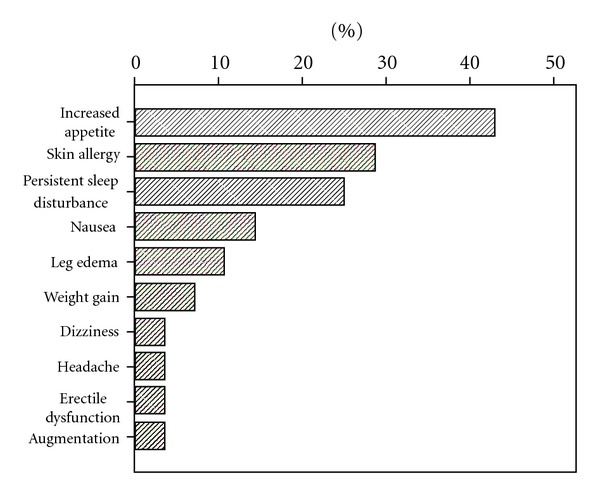
Adverse events.

An overview on the side-effects in this cohort is given in Figure 4. 71.4% had at least one adverse event, and 39.3% had two or more adverse events. Common side effects were nocturnal compulsive eating (42.9%), allergic skin reaction (28.6%), nausea (14.3%), and leg edema (10.7%). 25% of the patients complained about persistent problems in falling asleep and staying asleep despite fully satisfactory reduction of RLS symptoms. Augmentation occurred in one patient and led to termination of the drug. Two additional patients withdrew from rotigotine because of intolerable skin reactions.

## 4. Discussion

In this structured observation of 28 patients with severe RLS and severe augmentation under previous combined dopaminergic therapy, we found that rotigotine significantly reduces RLS severity and depressive symptoms, but is less effective regarding reduction of daytime sleepiness.

The median rotigotine dose was 4 mg, which is higher than in a previous large dose-finding trial, but consistent with the observations of the 12-month open label study [25, 27]. In 96.4% of the patients, a clinically significant reduction of RLS severity could be observed, in 70.4% symptoms were almost completely relieved. After 12 months at a stable dose, still, more than 50% of the patients were almost free of RLS symptoms, indicating a good long-term efficacy. However, it could be seen that in the course of 12 months after switch to rotigotine, mean IRLS scores tended to increase, suggesting a potential development of tolerance in the future. Further follow-up examinations will be needed to examine the response to rotigotine treatment in the course of several years.

Augmentation is a major limiting factor in long-term dopaminergic treatment. In this observation, although all subjects had suffered augmentation under their previous therapies, only one patient developed augmentation. Most interestingly, despite instruction, this one patient switched rotigotine doses on a daily basis, which may have led to varying plasma levels and may thus have triggered augmentation. Results are consistent with the finding of the prior large open-label trial, where augmentation was not a reported symptom [27]. 

Other side-effects of rotigotine were common in this cohort, more than 70% suffered at least one adverse event. However, generally, these were mild and did not lead to termination of the drug. Surprisingly, the most common adverse event, increased appetite with primarily nocturnal compulsive eating, was reported by almost half of the patients. Given that this side effect has not been described in previous treatment trials with rotigotine in RLS, it may be an issue limited to very severely affected patients with augmentation. Further studies are needed to substantiate this observation. Still, the fact that 24 of the 28 patients stayed on the medication over the 12-month period implies that rotigotine is well tolerable to patients with severe RLS. 

In addition to the positive effect of rotigotine on RLS symptoms, also a significant reduction of depressive symptoms was found in the majority of patients, underlining previous findings suggesting antidepressant efficacy of rotigotine [33]. The magnitude of the antidepressant effect was independent of the degree of reduction of RLS symptoms and could not be seen at the first follow-up examination, suggesting a delay in the response of depressive symptoms of at least 4 weeks in this cohort.

Another interesting finding is that 25% of the patients reported persistent difficulties in falling asleep and/or staying asleep despite fully satisfactory relief from RLS symptoms. This is also reflected by the finding that ESS scores as an indicator for daytime sleepiness were not altered during 12-month treatment with rotigotine, which contradicts data from previous studies demonstrating improvement in sleep and sleep-quality related scores [25, 26]. 

One limitation of this observation is its open-label character; however, the largest available trial on the long-term treatment was also open-label and showed results which are consistent with both findings of previous randomized controlled trials and results of our observation. 

In summary, rotigotine, which has proven effectiveness in mild, moderate, and severe RLS in previous studies, constitutes also an effective treatment for severe RLS with augmentation, and prior frustrating dopaminergic treatment. In these patients, in the clinical setting rotigotine effectively reduces RLS symptoms, augmentation, and depressive symptoms. Side effects are common, but usually of minor severity compared to the benefit. This observation underlines the potential of rotigotine in the treatment of RLS by extending the positive results found in previous randomized controlled trials and open-label trials to patients that could not be included in these trials due to severity of RLS symptoms. However, despite these promising results much longer follow-up periods are needed to confirm the value of rotigotine as loss of efficacy and augmentation may still occur years after treatment initiation.

##  Authors' Contributions

J. Godau and D. Berg designed the survey. J. Godau conducted and D. Berg supervised the study. J. Godau performed the statistical analysis and wrote up the first draft of the manuscript. D. Berg revised the manuscript and both authors approved the final version.
